# Prussian Blue Analogue-Derived p-n Junction Heterostructure for Photothermal Reverse Water–Gas Shift: Enhanced Activity and Selectivity via Synergistic Effects

**DOI:** 10.3390/nano15120904

**Published:** 2025-06-11

**Authors:** Shaorui Jia, Xinbo Zhang, Junhong Ma, Chaoyun Ma, Xue Yu, Yuanhao Wang

**Affiliations:** 1 State Key Laboratory of Chemistry and Utilization of Carbon Based Energy Resources, School of Chemical Engineering and Technology, Xinjiang University, Urumqi 830017, China; 107552201276@stu.xju.edu.cn (S.J.); 107552301253@stu.xju.edu.cn (X.Z.); mcy@xju.edu.cn (C.M.); yuxue@xju.edu.cn (X.Y.); 2 School of Future Technology, Xinjiang University, Urumqi 830017, China

**Keywords:** photothermal catalysis, CO_2_ hydrogenation, Prussian blue analogues-based catalyst, heterostructure, CO production

## Abstract

Photothermal catalytic CO_2_ conversion into chemicals that provide added value represents a promising strategy for sustainable energy utilization, yet the development of highly efficient, stable, and selective catalysts remains a significant challenge. Herein, we report a rationally designed p-n junction heterostructure, T-CZ-PBA (SC), synthesized via controlled pyrolysis of high crystalline Prussian blue analogues (PBA) precursor, which integrates CuCo alloy, ZnO, N-doped carbon (NC), and Zn^II^-Co^III^PBA into a synergistic architecture. This unique configuration offers dual functional advantages: (1) the abundant heterointerfaces provide highly active sites for enhanced CO_2_ and H_2_ adsorption/activation, and (2) the engineered energy band structure optimizes charge separation and transport efficiency. The optimized T-C_3_Z_1_-PBA (SC) achieves exceptional photothermal catalytic performance, demonstrating a CO_2_ conversion rate of 126.0 mmol gcat⁻^1^ h⁻^1^ with 98.8% CO selectivity under 350 °C light irradiation, while maintaining robust stability over 50 h of continuous operation. In situ diffuse reflectance infrared Fourier transform spectroscopy (in situ DRIFTS) investigations have identified COOH* as a critical reaction intermediate and elucidated that photoexcitation accelerates charge carrier dynamics, thereby substantially promoting the conversion of key intermediates (CO_2_* and CO*) and overall reaction kinetics. This research provides insights for engineering high-performance heterostructured catalysts by controlling interfacial and electronic structures.

## 1. Introduction

Transforming carbon dioxide into valuable chemicals through catalytic hydrogenation is a pivotal strategy for mitigating greenhouse gas emissions and repurposing CO_2_ [1,2,3,4]. The reverse water–gas shift (RWGS) reaction, which converts CO_2_ and H_2_ into CO and H_2_O, stands out as a particularly promising pathway for this transformation [5]. However, CO_2_’s inherent thermodynamic stability hinders its activation and conversion, necessitating high energy input, especially in the endothermic RWGS reaction [6,7]. Recently, photothermal catalytic technology has developed as an innovative approach for CO_2_ transformation [8,9,10]. It can simultaneously utilize solar energy and thermal energy, featuring high energy utilization efficiency and effectively enhancing the rate of catalytic reactions, which provides the possibility of achieving RWGS reaction under mild conditions.

From a materials design perspective, an ideal photothermal catalyst should satisfy multiple essential criteria: (1) broad-spectrum light absorption covering ultraviolet–visible–near-infrared regions to maximize solar energy utilization; (2) efficient photogenerated charge separation and transport enabled by optimized band structure and carrier regulation; and (3) prominent thermal effects (e.g., localized surface plasmon resonance or hot electron injection) to lower reaction activation energy. Furthermore, such catalysts must incorporate both thermal catalytic and photocatalytic active sites while maintaining robust thermal stability. Given that different material classes exhibit distinct advantages in fulfilling these requirements, the implementation of multi-component synergistic systems emerges as a particularly effective strategy for achieving superior photothermal catalytic performance [11,12,13].

In recent years, Prussian blue analogues (PBAs) and their derivatives have emerged as highly promising candidates for light-driven and photothermal catalytic CO_2_ conversion [14,15]. This growing interest stems from their unique combination of advantageous properties, including structurally tunable frameworks, broad spectral absorption capabilities, cost-effectiveness, environmental compatibility, and the presence of multiple functional active sites [16,17,18,19]. These distinctive characteristics position PBAs as versatile and efficient catalytic materials for sustainable energy conversion applications. For instance, Ding et al. [19] systematically evaluated ten PBA-based catalysts and found that they exhibit excellent photocatalytic CO_2_-to-CO conversion, with cobalt-based PBAs showing particularly superior performance. Fang et al. [20] synthesized open-framework composite metal oxides (Ni-Co-Fe-TMO) using PBA precursors for photothermal catalytic RWGS. The resulting heterojunctions, formed by intimate contact between metal oxides, feature fully exposed active sites, enabling stable catalytic performance at 200 °C with CO and CH₄ production rates of 2.98 and 1.48 mmol g⁻^1^ h⁻^1^, respectively, even after 12.5 h. Additionally, Co_2_C nanoparticles derived from Co-based PBAs has also been proven to exhibit decent performance in the thermal catalytic RWGS, which exhibits a CO_2_ conversion rate of 65.3 mmol gcat^−1^ h^−1^ with CO selectivity of 85% at 300 °C [21]. These findings indicate that cobalt-based PBA and its derivatives demonstrate promising potential in photo and/or thermal RWGS applications, but their CO selectivity, especially under photothermal catalytic conditions, still needs to be further improved.

In this work, we present a rationally designed PBA-derived heterostructured material, T-CZ-PBA (SC), synthesized through controlled pyrolysis of highly crystalline Cu^II^Zn^II^-Co^III^PBA. The unique architecture, integrating CuCo alloy, ZnO, NC, and Zn^II^-Co^III^PBA, delivers exceptional photothermal catalytic performance in the RWGS reaction. The optimized T-C_3_Z_1_-PBA (SC) catalyst achieves an outstanding CO_2_ conversion rate of 126.04 mmol gcat⁻^1^ h⁻^1^ with near-complete CO selectivity (98.8%) at 350 °C. The hybrid interface enhances charge separation/transfer and promotes efficient CO_2_ and H_2_ activation, contributing to its superior activity and stability. Even after 50 h of continuous operation, the catalyst retains robust performance, sustaining CO selectivity > 98% and CO_2_ conversion exceeding 100 mmol g⁻^1^ h⁻^1^. Furthermore, in situ diffuse reflectance infrared Fourier transform spectroscopy (in situ DRIFTS) analysis unveils critical reaction intermediates, offering mechanistic insights into the high-performance photothermal RWGS process.

## 2. Materials and Methods

### 2.1. Materials

All chemical reagents utilized in this study were sourced from Aladdin Chemical Reagents Co., Ltd., Shanghai, China.

### 2.2. Preparation of Catalysts

**Figure 1:**
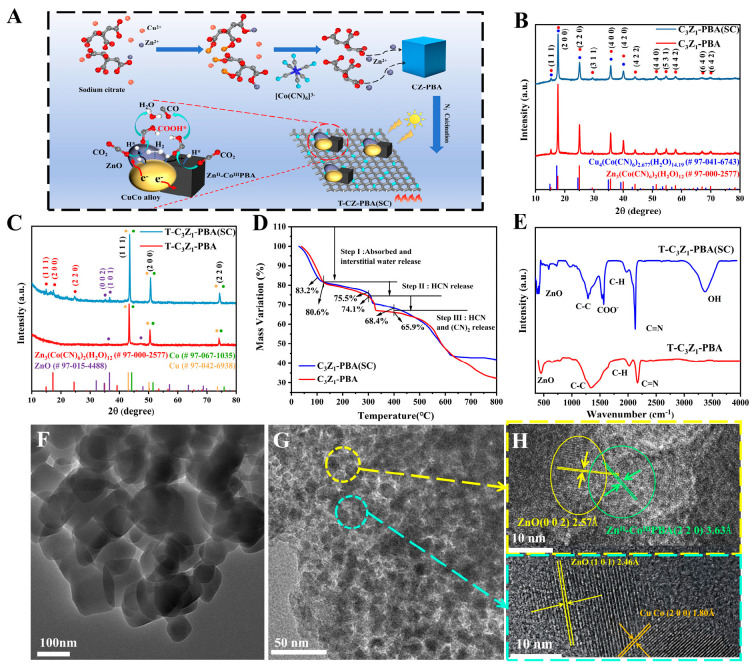
(**A**) Schematic illustration of T-C_x_Z_y_-PBA (SC) synthetic process. XRD patterns of (**B**) C_3_Z_1_-PBA (SC) and C_3_Z_1_-PBA, (**C**) T-C_3_Z_1_-PBA (SC) and T-C_3_Z_1_-PBA. (**D**) TGA of C_3_Z_1_-PBA (SC) and C_3_Z_1_-PBA. (**E**) FT-IR of T-C_3_Z_1_-PBA (SC) and T-C_3_Z_1_-PBA. (**F**) TEM image of C_3_Z_1_-PBA (SC). (**G**) TEM and (**H**) HRTEM images of T-C_3_Z_1_-PBA (SC).

As illustrated in Figure 1A, the catalysts were synthesized utilizing a two-step approach. Using T-C_3_Z_1_-PBA (SC) as a representative example, the detailed synthesis procedure can be outlined as follows.

(1) A solution designated as solution A was prepared by sequentially adding sodium citrate (C_6_H_5_Na_3_O_7_, 4.5 mmol), copper(II) nitrate trihydrate (Cu(NO_3_)_2_·3H_2_O, 4.5 mmol), and zinc(II) nitrate hexahydrate (Zn(NO_3_)_2_·6H_2_O, 1.5 mmol) to 100 mL of deionized water, ensuring complete dissolution. Concurrently, another solution, referred to as solution B, was created by dissolving tripotassium hexacyanocobaltate (K_3_[Co(CN)_6_], 2 mmol) in an additional 100 mL of deionized water. The two solutions were subsequently mixed thoroughly using magnetic agitation. Following a 20 h standing period, the resultant mixture was filtered and washed to yield the Cu^II^-Zn^II^-Co^III^PBA precursor, designated as C_3_Z_1_-PBA (SC), based on the molar ratio of copper to zinc.

(2) The C_3_Z_1_-PBA (SC) precursor was subjected to heating in a nitrogen atmosphere, reaching a temperature of 400 °C at a rate of 5 °C per minute over a duration of 4 h, resulting in the formation of T-C_3_Z_1_-PBA (SC). Utilizing a similar methodology, a series of T-C_x_Z_y_-PBA (SC) samples with varying copper and zinc proportions were synthesized. Additionally, corresponding C_x_Z_y_-PBA and T-C_x_Z_y_-PBA samples were produced without the inclusion of sodium citrate for comparative analysis. Furthermore, T-C-PBA (SC) and T-Z-PBA (SC) were prepared by exclusively introducing 6 mmol of either Cu(NO_3_)_2_·3H_2_O or Zn(NO_3_)_2_·6H_2_O, respectively, employing the aforementioned method.

### 2.3. Physical and Chemical Characterizations

The crystallographic characteristics of the samples were analyzed using X-ray diffraction (XRD) with Cu Kα radiation, employing a Rigaku instrument from Tokyo, Japan. Microscopic morphological data were acquired through transmission electron microscopy (TEM, S2403-F200, 200 k eV, JEOL, Beijing, China) and high-resolution transmission electron microscopy (HRTEM, S2403-F200, 200 k eV, JEOL, China), complemented by energy-dispersive X-ray spectroscopy (EDS, SUPER X, Beijing, China). The optical absorption properties were assessed using ultraviolet–visible diffuse reflectance spectroscopy (UV-vis DRS, Hitachi U-3010, Tokyo, Japan). Photoluminescence (PL) spectra were recorded with a Hitachi fluorescence spectrophotometer (F-4500, Tokyo, Japan). The surface chemical states of the catalysts were examined via X-ray photoelectron spectroscopy (XPS, Thermo ESCALAB 250Xi, Waltham, MA, USA). Additionally, Fourier transform infrared spectroscopy (FT-IR) analysis was conducted using a VERTEX 70 RAMI infrared spectrometer from the German company BRUKER (Ettlingen, Germany) to investigate the composition and structure of the catalyst. Thermogravimetric analysis (TGA) was performed using a HITACHI STA7300 instrument under a nitrogen atmosphere.

In situ diffuse reflectance infrared Fourier transform spectroscopy (in situ DRIFTS) measurements were carried out on a BRUKER FTIR spectrometer (INVENIO) equipped with a liquid nitrogen-cooled MCT detector. Prior to the measurements, the catalyst underwent a pretreatment under nitrogen at 150 °C for 10 min to eliminate moisture and adsorbed gases. A background spectrum was subsequently collected. Following this, in situ reaction tests were conducted by switching the gas flow to a mixture of H_2_, CO_2_, and N_2_ in a molar ratio of 48:12:40, with a total flow rate of 25 mL min^−1^.

### 2.4. Catalytic Tests

Initially, 0.06 g of catalyst is thoroughly combined with 0.4 g of quartz sand (40–60 mesh) and subsequently placed within the isothermal zone at the center of the reactor. A K-type thermocouple is employed to monitor the temperature of the catalyst bed throughout the process. During the photothermal catalytic reaction, a 300 W xenon light source is introduced laterally into the quartz pool; however, this light source is deactivated during the thermal reaction phase. The proportions of the reactant gases are regulated using a mass flow meter. Upon reaching the designated reaction temperature (250–350 °C) within a nitrogen atmosphere, the mixed feed gas, comprising H_2_/CO_2_/N_2_ in a ratio of 48/12/40 (with 40% nitrogen utilized as an internal standard), is introduced into the reactor at a gas hourly space velocity (GHSV) of 50,000 mL g⁻^1^ h⁻^1^. Analytical assessments are performed utilizing a gas chromatograph (GC9790plus, FULI, Wenling, Zhejiang, China) that is equipped with both a thermal conductivity detector (TCD) and a flame ionization detector (FID).

The photoelectric properties of the samples were evaluated using a CHI660 electrochemical workstation (Shanghai Chenhua Instruments Co., Ltd., Shanghai, China) configured with a three-electrode system. In this setup, a platinum wire served as the counter electrode, while an Ag/AgCl electrode (saturated with KCl) functioned as the reference electrode. The working electrode was prepared by first dispersing 10 mg of the catalyst sample ultrasonically in 1 mL of ethanol, supplemented with 50 μL of a 5% Nafion ethanol solution, to create a homogeneous suspension. Following this, 100 μL of the resulting suspension was applied to indium tin oxide (ITO) glass and allowed to dry at ambient temperature. The photocurrent was subsequently measured under visible light irradiation provided by a 300 W xenon lamp. Additionally, electrochemical impedance spectroscopy (EIS) measurements were conducted in a 0.1 M Na_2_SO_4_ aqueous solution, covering a frequency range from 10,000 Hz to 0.1 Hz.

## 3. Results and Discussion

The preparation process of the T-C_x_Z_y_-PBA (SC) series of samples is illustrated in Figure 1A. Benefiting from the strong chelation of sodium citrate and zinc ions [22,23], some highly crystalline Zn^II^-Co^III^PBA was formed in the pre-synthesized CZ-PBA (SC), which was retained and combined with the pyrolysis product CuCo-ZnO@NC during the subsequent calcination process to achieve heterojunction structure materials T-C_x_Z_y_-PBA (SC), where x:y represents the molar ratio of Cu to Zn.

XRD patterns of the typical samples are shown in Figure 1B,C. The C_3_Z_1_-PBA (SC) sample synthesized in the presence of sodium citrate exhibits characteristic diffraction peaks corresponding to both Cu^II^-Co^III^PBA and Zn^II^-Co^III^PBA phases. Notably, these diffraction patterns are structurally similar to those of the control C_3_Z_1_-PBA sample prepared without sodium citrate addition (Figure 1B). But after the following heat treatment, they transformed into T-C_3_Z_1_-PBA (SC) and T-C_3_Z_1_-PBA with distinctly different structures (Figure 1C). The XRD pattern of T-C_3_Z_1_-PBA (SC) exhibits distinct diffraction peaks associated with metallic CuCo alloy and ZnO, along with the peaks at 15°, 17°, and 24° corresponding to the Zn^II^-Co^III^PBA structure, while the T-C_3_Z_1_-PBA only displays peaks related to CuCo alloy and ZnO. This result indicates that the Zn^II^-Co^III^PBA structural unit in C_3_Z_1_-PBA (SC) sample exhibits high thermal stability, which does not decompose during the pyrolysis process. TGA was further used to verify the crystallinity and thermal stability of the C_3_Z_1_-PBA (SC) and C_3_Z_1_-PBA. As shown in Figure 1D, the C_3_Z_1_-PBA (SC) contains only 16.8% crystalline water, lower than 19.4% of C_3_Z_1_-PBA, and the thermal weight loss rate of the former before 400 °C is also significantly lower than that of the latter. The results demonstrate that sodium citrate effectively stabilizes the PBA structure, allowing C_3_Z_1_-PBA (SC) to maintain some Zn^II^-Co^III^PBA structural characteristics post-calcination.

To further investigate the molecular structure and chemical composition of T-C_3_Z_1_-PBA (SC) and T-C_3_Z_1_-PBA, FT-IR was employed as depicted in Figure 1E. The characteristic peaks at 1283, 1970, and 2160 cm^−1^ in T-C_3_Z_1_-PBA correspond to the C-C, C-H, and C=N from NC material produced by decomposition of C_3_Z_1_-PBA [24]; meanwhile, the stretching vibration absorption peak of ZnO could be observed at 440 cm^−1^. It is noteworthy that two obvious absorption peaks at 2100 and 3360 cm^−1^, which could be assigned to -C≡N and crystalline water in PBA structure [19], appear in T-C_3_Z_1_-PBA (SC); this result is consistent with the XRD analysis. Concurrently, the signal of COO^-^ from pyrolysis of sodium citrate can be found at 1560 cm^−1^.

TEM was further used to investigate the morphology of the as-prepared samples. As illustrated in Figure 1F, the C_3_Z_1_-PBA (SC) precursor exhibits a predominantly cubic morphology, characteristic of the typical PBA structure. Following calcination, the resulting T-C_3_Z_1_-PBA (SC) material transforms into carbon-coated nanoparticles, as clearly demonstrated in Figure 1E. The HRTEM analyses of T-C_3_Z_1_-PBA (SC) are shown in Figure 1G, based on the HRTEM images obtained from different particle regions, well-defined lattice fringes can be clearly observed, demonstrating an intergrowth relationship between ZnO (002) and Zn^II^-Co^III^PBA (220) planes, as well as between ZnO (101) and CuCo alloy (200) planes, respectively. This will be conducive to providing more interfacial active sites and promoting catalytic reactions. Additionally, EDS analysis (Figure S1) confirms that the atomic percentages (at%) of Cu and Zn in T-C_3_Z_1_-PBA (SC) are 7.34% and 2.17%, respectively, consistent with the expected stoichiometric ratio of these elements in the sample.

**Figure 2:**
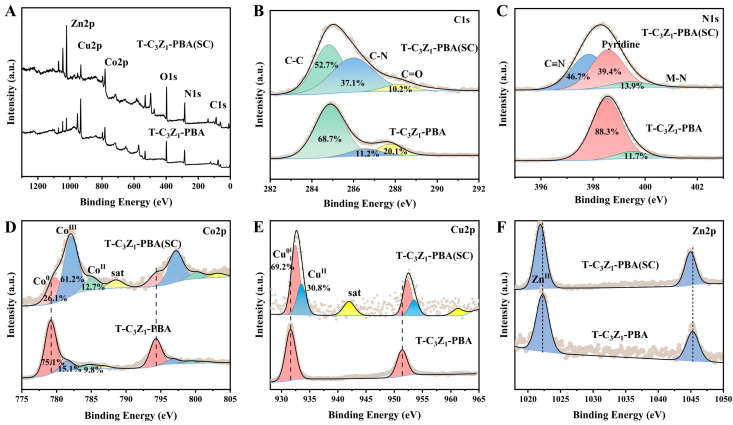
(**A**) XPS survey spectra. XPS high-resolution spectra for (**B**) C 1s, (**C**) N 1s, (**D**) Co 2p, (**E**) Cu 2p, and (**F**) Zn 2p of T-C_3_Z_1_-PBA (SC) and T-C_3_Z_1_-PBA.

XPS was further employed to investigate the surface composition of T-C_3_Z_1_-PBA (SC) and T-C_3_Z_1_-PBA, as illustrated in Figure 2. Comparative analysis of the deconvoluted C1s and N1s spectra reveals a significant increase in the relative content of C-N (ca. 286.1 eV) in T-C_3_Z_1_-PBA (SC) (Figure 2B). Additionally, a distinct C≡N characteristic peak emerges at 397.9 eV (Figure 2C), confirming the presence of the PBA structure in this sample [25]. The high-resolution Co 2p spectrum (Figure 2D) exhibits peaks at 779.2, 781.7, and 784.9 eV, corresponding to Co^0^, Co^3+^, and Co^2+^ species, respectively. Owing to the Zn^II^-Co^III^PBA structure in T-C_3_Z_1_-PBA (SC), the proportion of Co^3+^ in this sample is markedly higher than in T-C_3_Z_1_-PBA (61.2% vs. 15.1%). Furthermore, compared with T-C_3_Z_1_-PBA, the XPS peaks of Cu^0^ and Co^0^ in T-C_3_Z_1_-PBA (SC) exhibit negative shifts, while Zn^2+^ shows a positive shift (Figure 2D–F). This observation suggests enhanced electron transfer from the CuCo alloy to Zn^2+^ in the latter. Such electron redistribution likely creates an electron-rich state on Zn^II^-Co^III^PBA (or ZnO) in T-C_3_Z_1_-PBA (SC), thereby promoting CO_2_ adsorption and activation [26].

**Figure 3:**
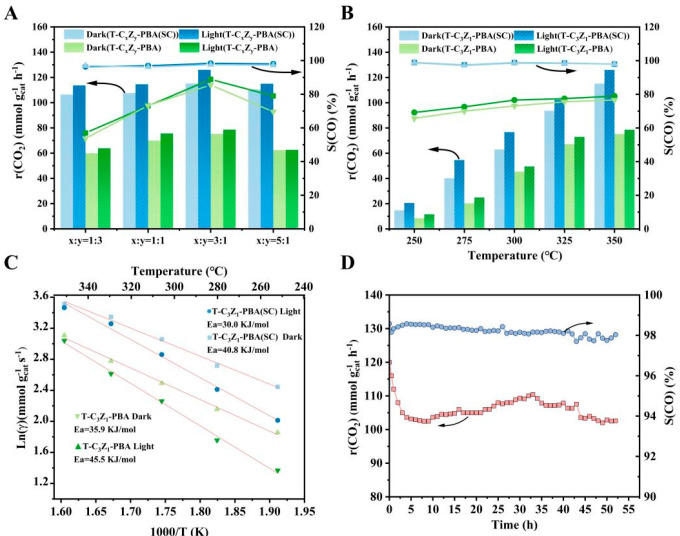
Thermal and photothermal catalytic conversion rates of CO_2_ and CO selectivities of (**A**) T-C_x_Z_y_-PBA (SC) and T-C_x_Z_y_-PBA samples at 350 °C and (**B**) T-C_3_Z_1_-PBA (SC), T-C_3_Z_1_-PBA at different temperatures. (**C**) Arrhenius plots under dark and light conditions of T-C_3_Z_1_-PBA (SC) and T-C_3_Z_1_-PBA. (**D**) Stability tests of T-C_3_Z_1_-PBA (SC) at 350 °C under light conditions.

The results of the photothermal catalysis CO_2_RR test of the catalyst are shown in Figure 3A. At 350 °C, the T-C_x_Z_y_-PBA (SC) series of samples exhibit significant enhanced catalytic activity and selectivity for the RWGS reaction compared to the corresponding T-C_x_Z_y_-PBA in both thermal and photothermal conditions. Particularly, the T-C_3_Z_1_-PBA (SC) exhibits optimal catalytic behavior, achieving a CO_2_ conversion rate of 126.04 mmol g_cat_^−1^ h^−1^ with light irradiation, 12 mmol g_cat_^−1^ h^−1^ higher than that of the corresponding thermal catalysis, and its CO selectivity is up to 98.8%. Figure 3B shows the catalytic CO_2_ hydrogenation performances over T-C_3_Z_1_-PBA (SC) and T-C_3_Z_1_-PBA as functions of reaction temperature. The corresponding apparent activation energy (Ea) values calculated based on the Arrhenius equation are displayed in Figure 3C. It is evident that the Ea value for T-C_3_Z_1_-PBA (SC) is considerably lower than that for T-C_3_Z_1_-PBA, regardless of whether light is applied. The addition of light led to a certain degree of decrease in the Ea for both samples, with a more pronounced reduction for T-C_3_Z_1_-PBA (SC). From these results, it can be inferred that the additional interface sites generated from coexistence of Zn^II^-Co^III^PBA and CuCo-ZnO@NC in T-C_3_Z_1_-PBA (SC) play a crucial role in enhancing both the catalytic activity and selectivity for RWGS reaction.

To verify the potential synergism between the Cu and Zn species, we also evaluated the catalytic performance of the as-prepared T-C-PBA (SC) and T-Z-PBA (SC) samples (Figure S2). Within the investigated temperature range, the CO_2_ conversion rates of both samples are lower than those of the corresponding sample T-C_3_Z_1_-PBA (SC), indicating the important role of the synergy between the Cu- and Zn-based components in improving the catalytic performance. The stability test result for the optimized T-C_3_Z_1_-PBA (SC) catalyst at 350 °C with illumination is presented in Figure 3D. After 52 h of continuous operation, the CO_2_ conversion rate can still sustain above 100 mmol g_cat_^−1^ h^−1^, and the selectivity of the CO product remains at ~98%, indicating the commendable catalytic performance over extended periods of use. The superiority of T-C_3_Z_1_-PBA (SC) can be clearly seen by comparing the CO_2_ conversion rate and CO selectivity of transition metal-based catalysts in recent reports for photothermal catalytic CO_2_ hydrogenation (Table S1).

The surface properties of the prepared samples were studied using Mott Schottky impedance testing, as depicted in Figure S3. The impedance curve of T-C_3_Z_1_-PBA exhibits a positive slope, indicative of an n-type semiconductor behavior [27]. In contrast, T-C_3_Z_1_-PBA (SC) displays both positive and negative slope curves, suggesting the presence of p-n heterojunctions within the sample, where the p-type structure should be related to the PBA structure retained in this sample. This p-n junction structure would augment the material’s charge transfer capability, thus enhancing both the photocatalytic and thermocatalytic behaviors simultaneously.

**Figure 4:**
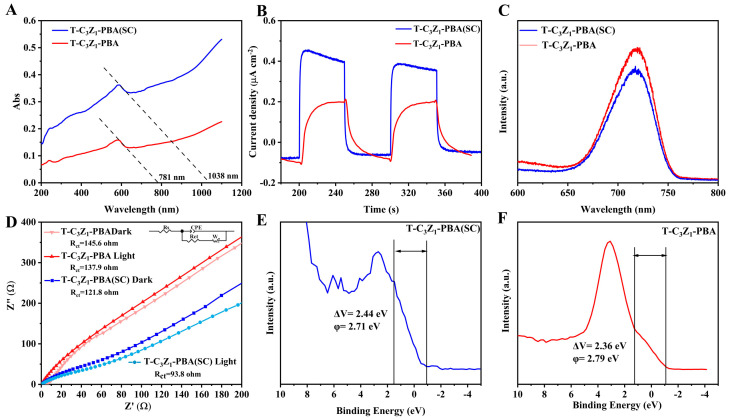
(**A**) UV-vis DRS spectra of T-C_3_Z_1_-PBA (SC) and T-C_3_Z_1_-PBA. (**B**) Photocurrent curves, (**C**) PL spectra, and (**D**) EIS plots of T-C_3_Z_1_-PBA (SC) and T-C_3_Z_1_-PBA. Work functions of (**E**) T-C_3_Z_1_-PBA (SC) and (**F**) T-C_3_Z_1_-PBA.

The optical properties of semiconductors are closely related to their energy band structure [28]. The test result of UV-vis DRS is shown in Figure 4A. The absorption edges of T-C_3_Z_1_-PBA (SC) and T-C_3_Z_1_-PBA are located at 1038 nm and 781 nm respectively, implying their good visible light responsiveness. The absorption edge of T-C_3_Z_1_-PBA (SC) shifts toward longer wavelength regions, illustrating the boosted sunlight utilization efficiency [29]. The bandgap energy (Eg) of T-C_3_Z_1_-PBA (SC) and T-C_3_Z_1_-PBA determined from semiconductor formula (αhν)^n^ = k(hν − E_g_) are 1.64 and 1.88 eV, respectively (Figure 4B and Figure S4A). The narrower band gap of T-C_3_Z_1_-PBA (SC) is attributable to the presence of the p-n junction in it, which is believed to contribute to the ease of electron–hole pair formation and broaden the spectrum of absorbed light [28]. By employing the Mott–Schottky equation [30,31], the conduction band potential (E_CB_) values of T-C_3_Z_1_-PBA (SC) and T-C_3_Z_1_-PBA were calculated to be −0.559 and −0.542 eV, respectively (Figure S5), both of which are negative than −0.53 eV, facilitating the reduction of CO_2_ to CO [32]. Additionally, comparative analysis reveals that T-C_3_Z_1_-PBA (SC) exhibits enhanced photocurrent response under visible light illumination (Figure 4B) and substantially reduced PL intensity (Figure 4C) relative to its T-C_3_Z_1_-PBA counterpart. These observations collectively demonstrate the effective suppression of charge recombination in T-C_3_Z_1_-PBA (SC), consequently resulting in superior charge separation efficiency. The EIS Nyquist plots under dark and light conditions are shown in Figure 4D; the smaller impedance arc of T-C_3_Z_1_-PBA (SC) indicates that it facilitates faster charge transfer. When exposed to light, both samples showed improved electron transfer, as evidenced by their lower charge transfer resistance (Rct). Remarkably, T-C_3_Z_1_-PBA (SC) exhibited the most dramatic Rct reduction, indicating its exceptional charge-transfer efficiency in delivering photogenerated carriers to active sites, thus optimizing their utilization in catalytic processes [33]. It should be noted that while the localized surface plasmon resonance (LSPR) effect of the CuCo alloy in both samples can elevate the local surface temperature of the catalyst under illumination, thereby accelerating reaction kinetics, the significantly enhanced catalytic activity of T-C_3_Z_1_-PBA (SC) under light irradiation cannot be explained solely by this thermal effect [34]. Instead, we attribute the exceptional performance primarily to the unique p-n junction structure in T-C_3_Z_1_-PBA (SC). This structure substantially improves the separation, migration, and surface reaction efficiency of photogenerated carriers through energy band structure modulation as discussed above. Consequently, the superior catalytic activity under light conditions of T-C_3_Z_1_-PBA (SC) arises from the synergistic interplay between plasmonic heating and the engineered p-n junction. Furthermore, the work function (Δϕ) of T-C_3_Z_1_-PBA (SC) and T-C_3_Z_1_-PBA were calculated from the formula ΔV = Δϕ − φ using VB-XPS data (Figure 4E,F). Where ΔV is the contact potential difference, represented by the distance between the two inflection points of the curve, and φ is the instrument work function (5.15 eV). The Δϕ of T-C_3_Z_1_-PBA (SC) and T-C_3_Z_1_-PBA were determined to be 2.98 and 3.48 eV, respectively. The reduced work function of T-C_3_Z_1_-PBA (SC) enhances its electron donation to adsorbed CO_2_, leading to a more efficient CO_2_ reduction process [35,36].

**Figure 5:**
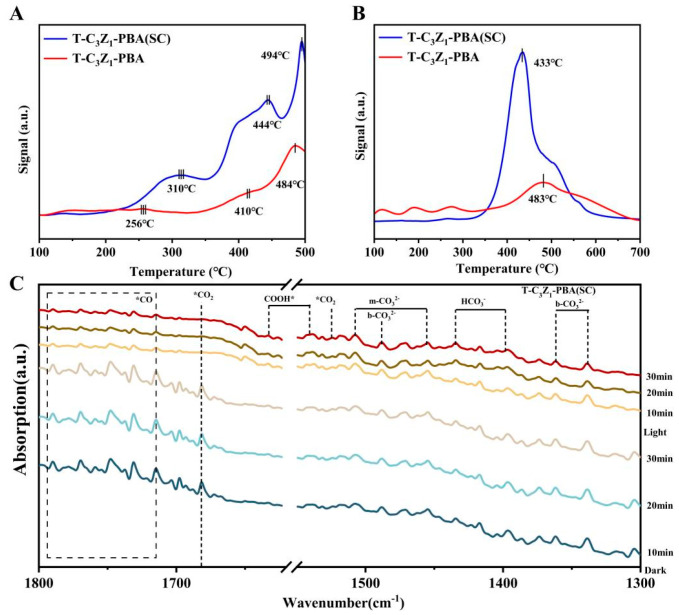
(**A**) CO_2_-TPD and (**B**) H_2_-TPR profiles of T-C_3_Z_1_-PBA (SC) and T-C_3_Z_1_-PBA. (**C**) In situ DRIFTS spectra under dark and light conditions of T-C_3_Z_1_-PBA (SC).

CO_2_ temperature-programmed desorption (CO_2_-TPD) technology was used to explore the interaction between CO_2_ molecules and the prepared catalysts, as shown in Figure 5A. For both the T-C_3_Z_1_-PBA (SC) and T-C_3_Z_1_-PBA samples, there are multiple desorption peaks appearing in the temperature range of 250–500 °C, representing the chemisorption between catalysts and CO_2_ [37]. For T-C_3_Z_1_-PBA, the peaks at approximately 256 and 410 °C could be associated with moderately chemisorbed CO_2_, whereas the peak centered at 484 °C is related to the desorption of strongly chemically bonded CO_2_ molecules. Notably, T-C_3_Z_1_-PBA (SC) exhibits a higher and intensified CO_2_ desorption temperature at 310, 444, and 494 °C, illustrating that the special hybrid-structure interface in T-C_3_Z_1_-PBA (SC) could provide abundant strong adsorption sites for activating CO_2_ molecules [38].

Hydrogen activation is pivotal in the hydrogenation reactions of carbon-containing substances, influencing CO_2_ conversion rates and product selectivity. The hydrogen temperature-programmed reduction (H_2_-TPR) was used to evaluate the hydrogen activation ability of catalytic samples. As displayed in Figure 5B, a main broader and unsymmetrical peak centered at ~483 °C can be found in the T-C_3_Z_1_-PBA sample, which could be attributed to the reduction of Zn^2+^. Compared to T-C_3_Z_1_-PBA, a more pronounced peak centered at 433 °C appears in the curve of T-C_3_Z_1_-PBA (SC), indicating the enhanced dissociation and spillover of H_2_ [38,39].

In situ DRIFTS was performed to delve deeper into the possible reaction mechanisms of CO_2_RR on T-C_3_Z_1_-PBA (SC). As demonstrated in Figure 5C, under both thermal and photothermal conditions, the similar absorption peaks of *CO_2_ at 1522 and 1685 cm^−1^, monodentate carbonate groups (m-CO_3_^2−^) at 1459 and 1509 cm^−1^, bidentate carbonates (b-CO_3_^2−^) at 1336, 1359, and 1490 cm^−1^, and bicarbonate groups (HCO_3_^−^) at 1401 and 1469 cm^−1^ can be observed 10 min after the introduction of CO_2_ and H_2_, and as time continues to extend, the intensities of these peaks remain unchanged, indicating that the reaction has reached a stable state [39,40,41]. It is noteworthy that the coordination activation of CO_2_ on the surface of T-C_3_Z_1_-PBA (SC) is indicated by the presence of COOH* at 1637 cm^−1^, which is a key intermediate for the selective catalytic reduction of CO_2_ to CO [42]; meanwhile, the CO* could also be observed at 1700–1800 cm^−1^. These findings suggest that the plausible CO_2_RR pathway on the T-C_3_Z_1_-PBA (SC) is as follows:

CO_2_ (g) + * → CO_2_*

CO_2_* + H^+^ + e^−^ → COOH*

COOH* + H^+^ + e^−^ → CO* + H_2_O

CO* → CO (g) + *

Notably, the addition of light results in the attenuation of intensity for reaction intermediates, especially CO_2_* and CO*, indicating that photogenerated electrons effectively promote the conversion of these intermediates, thus accelerating the reaction process.

## 4. Conclusions

In summary, a novel PBA-derived hybrid-structure catalyst, T-CZ-PBA (SC), consisting of Zn^II^-Co^III^PBA, CuCo alloy, ZnO, and NC, was successfully constructed for an RWGS reaction. Based on the effective charge separation and transfer originating from this special heterostructure, as well as the strong adsorption/activation of CO_2_ and H_2_, the optimized T-C_3_Z_1_-PBA (SC) exhibits 126.0 mmol g_cat_^−1^ h^−1^ of CO_2_ conversion capability and 98.8% selectivity of the CO product at 350 °C with irradiation. In situ DRIFTS indicated that the light accelerates the conversion of intermediates like CO_2_* and CO*, thus boosting the catalytic activity. This work will effectively progress the research in the controllable preparation of highly efficient hybrid-structure photothermal catalysts.

## Data Availability

The data will be made available upon request.

## Supplementary Materials

The following supporting information can be downloaded at https://www.mdpi.com/article/10.3390/nano15120904/s1: Figure S1: The EDS spectra of T-C_3_Z_1_-PBA (SC); Figure S2: Photothermal and thermal catalytic conversion rates of CO_2_ and CO selectivities of T-Z-PBA (SC) and T-C-PBA (SC) at different temperature; Figure S3: M-S plots of (A) T-C_3_Z_1_-PBA and (B) T-C_3_Z_1_-PBA (SC); Figure S4: Tauc curves of (A) T-C_3_Z_1_-PBA and (B) T-C_3_Z_1_-PBA (SC); Figure S5: E_CB_ of (A) T-C_3_Z_1_-PBA and (B) T-C_3_Z_1_-PBA (SC); Table S1: The comparison of catalytic activities with other reported catalysts for photo-thermal catalytic CO_2_ reduction. Refs. [42,43,44,45,46,47,48,49,50,51] are cited in the supplementary materials.
