# Supplementary material for: Prussian Blue Analogue-Derived p-n Junction Heterostructure for Photothermal Reverse Water–Gas Shift: Enhanced Activity and Selectivity via Synergistic Effects
Source: Nanomaterials (Basel). 2025 Jun 11;15(12):904. doi: 10.3390/nano15120904 (PMC12195841; doi:10.3390/nano15120904)
Supplement: Supplementary file 1 [file nanomaterials-15-00904-s001.zip › nanomaterials-3663913-supplementary.pdf]

# Supplementary Materials

## Figures

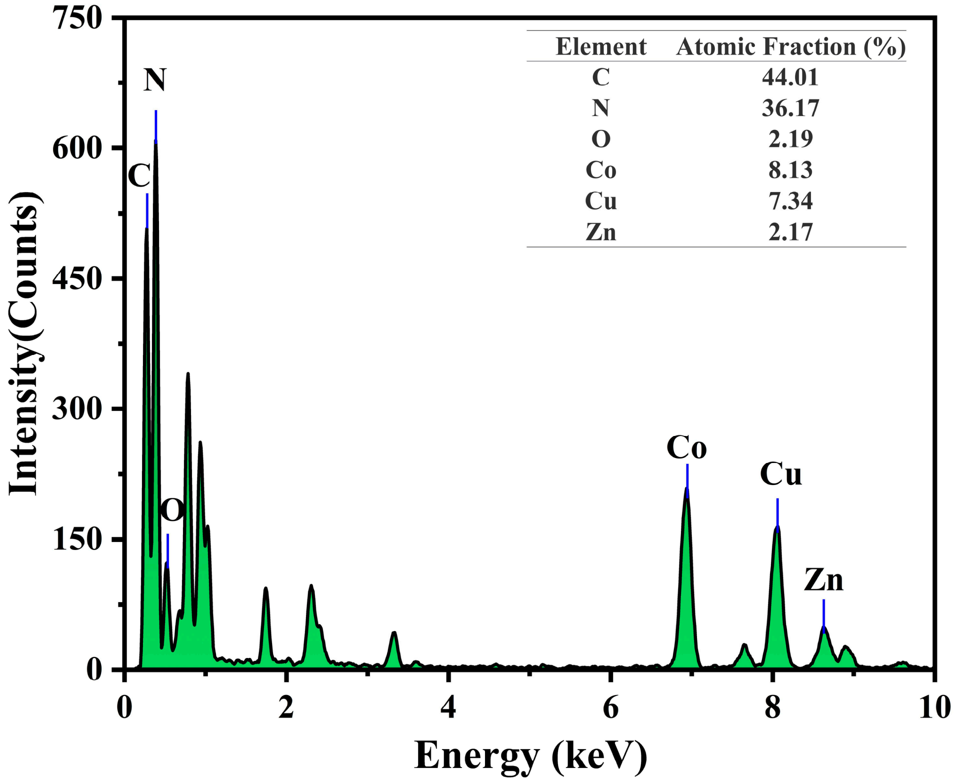


**Figure S1.** The EDS spectra of T-$C_3Z_1$-PBA (SC).

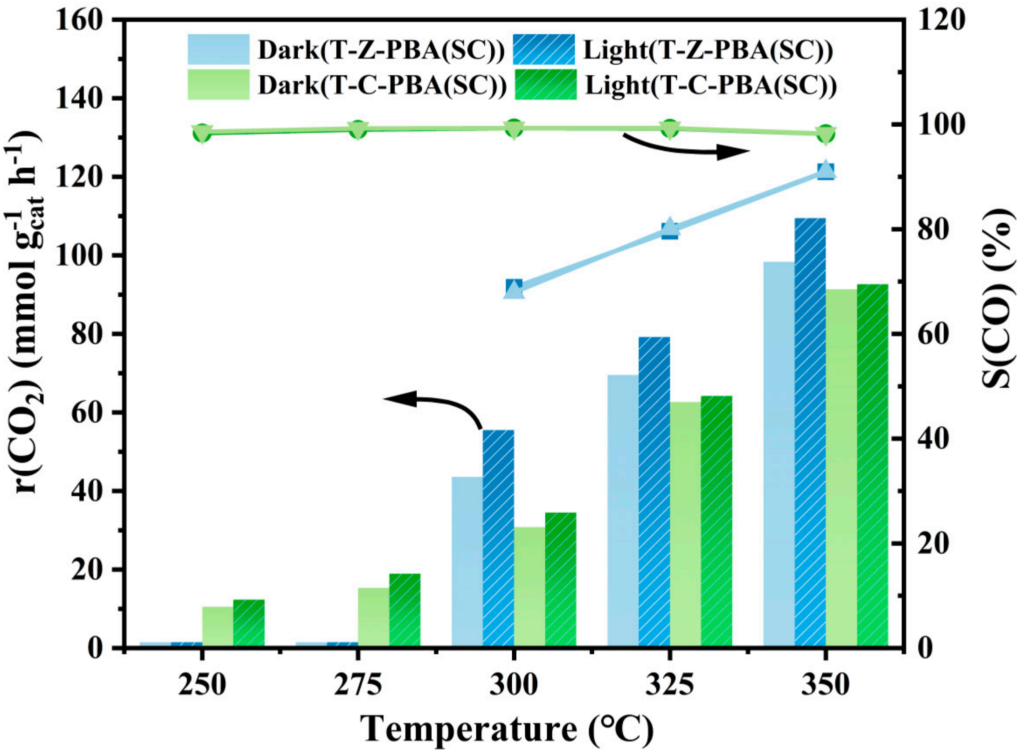


**Figure S2.** Photothermal and thermal catalytic conversion rates of $CO_2$ and CO selectivities of T-Z-PBA (SC) and T-C-PBA (SC) at different temperatures.

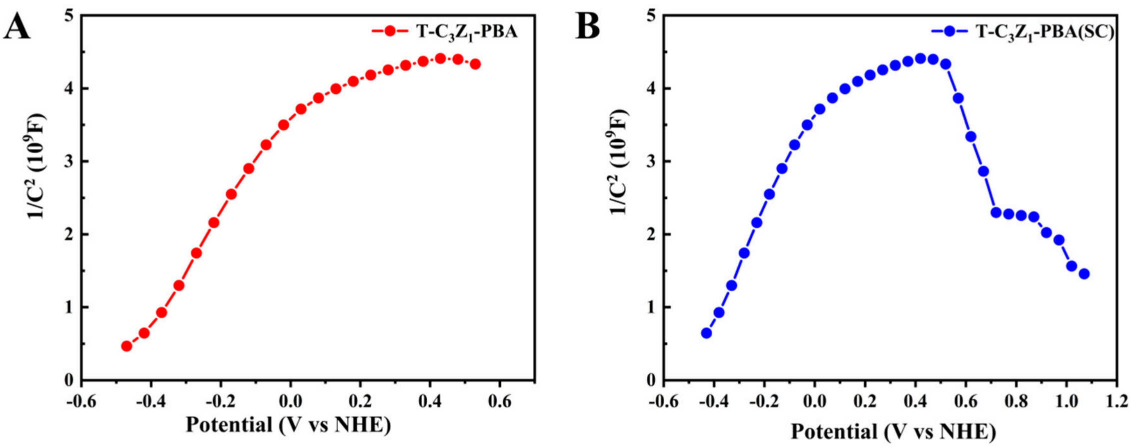


**Figure S3.** M-S plots of (A) T-$C_3Z_1$-PBA and (B) T-$C_3Z_1$-PBA (SC).

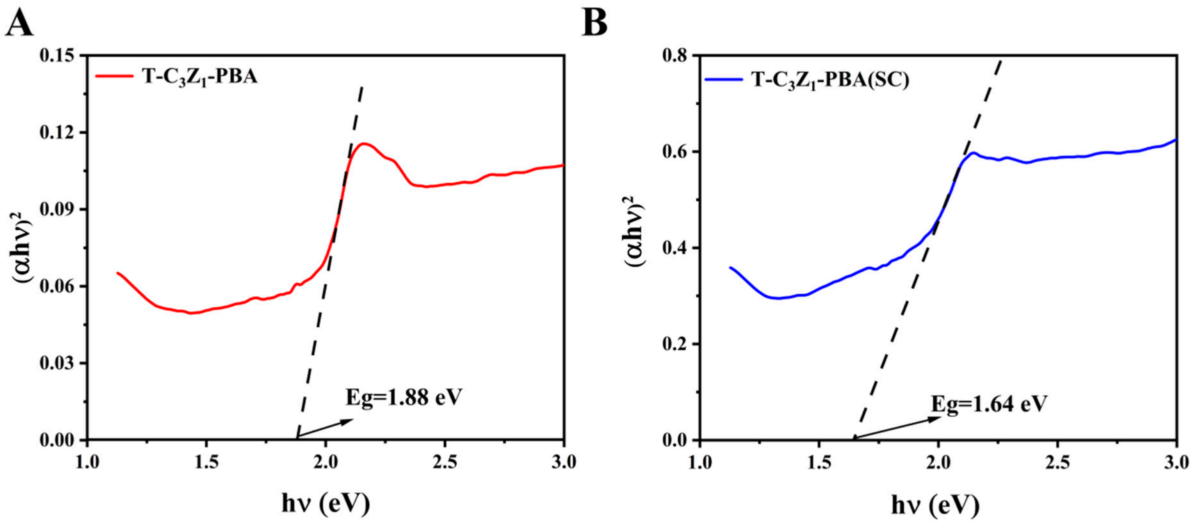


**Figure S4.** Tauc curves of (A) T-$C_3Z_1$-PBA and (B) T-$C_3Z_1$-PBA (SC).

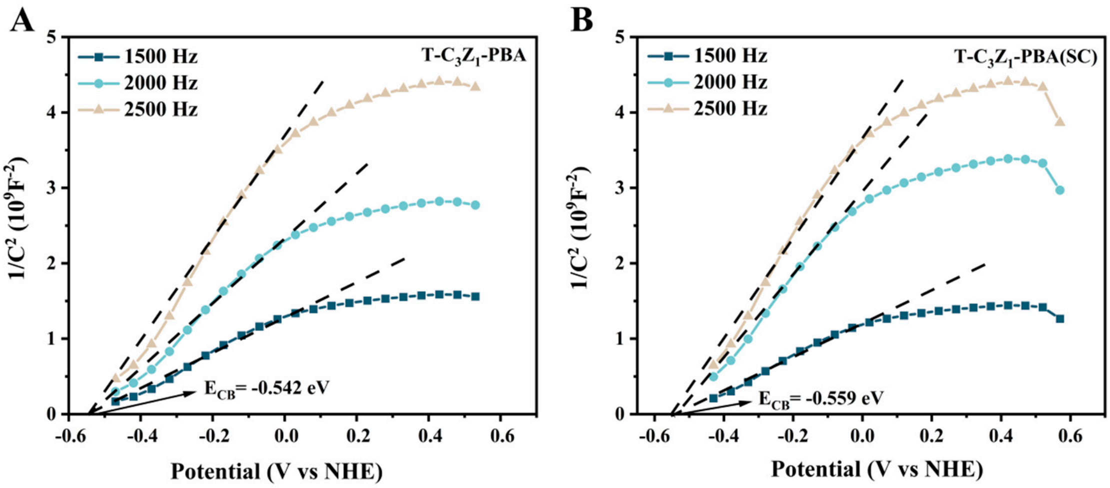


**Figure S5.** $E_{CB}$ of (A) T-$C_3Z_1$-PBA and (B) T-$C_3Z_1$-PBA (SC).

**Table S1** The comparison of catalytic activities with other reported catalysts for photo-thermal catalytic $CO_2$ reduction.

| Catalysts | Reaction conditions | $CO_2$ rate ($mmol\cdot g_{cat}^{-1}\cdot h^{-1}$) | CO selectivity (%) | Ref. |
|---|---|---|---|---|
| T-$C_3Z_1$-PBA(SC) | 300 W Xe lamp, 2 W $m^{-2}$,300℃ | 77.66 | 98.8 | This work |
| Cu-$CeO_2$-NR | 44.6 mW $cm^{-2}$, 328℃ | 22.74 | 99% | [S1] |
| Pt/30-CDs/Zr-MOF. | 1.2 W $cm^{-2}$,300℃ | 22.66 | 99% | [S2] |
| $Co_{15}$HAP | 1080 W $m^{-2}$,300℃ | 35.56 | 90 | [S3] |
| $In_2O_{3-x}/In_2O_3$ | 0.8 W $cm^{-2}$,300℃ | 2.05 | 100 | [S4] |
| Cu-HAP | LED light , 2 W $cm^{-2}$,300℃ | 0.75 | 99.73 | [S5] |
| $K^+$-Co-C | 300 W Xe lamp, 2.8 W $cm^{-2}$,300℃ | 36.7 | 100 | [S6] |
| c-$TiO_2$@a-$TiO_{2-x}(OH)_y$ | 0.8 W $cm^{-2}$,300℃ | 0.65 | 100 | [S7] |
| $CuCo_5BO_x$ | 500 mW $cm^{-2}$, 300℃ | 93.47 | 98 | [S8] |
| $Ni_{12}P_5/SiO_2$ | 0.8 W $cm^{-2}$,300℃ | 13.5 | 100 | [S9] |
| $Co_7Cu_1Mn_1O_x$(200) | 300 W Xe lamp, 300℃ | 25 | 30 | [S10] |

# Supplementary references

(1) Li, H.; Xiao, Z.; Hao, R.; Tan, X.; Ye, F.; Gu, J.; Li, J.; Li, G.; Zou, J.; Wang, D. Encapsulating carbon quantum dots by Zr-MOF-supported Pt nanoparticles for enhanced photothermal RWGS reaction. *Separation and Purification Technology* **2025**, *365*, 132637.

(2) Guo, J.; Zhang, X.; Wang, Y.; Liao, L.; Xie, Q.; Mo, S. Constructing asymmetric Cu-Ce site pairs over defective Cu/$CeO_2$ catalysts for efficient concentrating solar-driven photothermal RWGS reaction. *Journal of Alloys and Compounds* **2025**, *1024*, 180228.

(3) Peng, Y.; Szalad, H.; Nikacevic, P.; Gorni, G.; Goberna, S.; Simonelli, L.; Albero, J.; López, N.; García, H. Co-doped hydroxyapatite as photothermal catalyst for selective $CO_2$ hydrogenation. *Applied Catalysis B: Environment and Energy* **2023**, *333*, 122790.

(4) Wang, L.; Dong, Y.; Yan, T.; Hu, Z.; Ali, F. M.; Meira, D. M.; Duchesne, P. N.; Loh, J. Y. Y.; Qiu, C.; Storey, E. E.; et al. Black indium oxide a photothermal $CO_2$ hydrogenation catalyst. *Nature Communications* **2020**, *11*, 2432.

(5) Guo, J.; Duchesne, P. N.; Wang, L.; Song, R.; Xia, M.; Ulmer, U.; Sun, W.; Dong, Y.; Loh, J. Y. Y.; Kherani, N. P.; et al. High-Performance, Scalable, and Low-Cost Copper Hydroxyapatite for Photothermal $CO_2$ Reduction. *ACS Catalysis* **2020**, *10*, 13668-13681.

(6) Wang, H.; Fu, S.; Shang, B.; Jeon, S.; Zhong, Y.; Harmon, N. J.; Choi, C.; Stach, E. A.; Wang, H. Solar-Driven $CO_2$ Conversion via Optimized Photothermal Catalysis in a Lotus Pod Structure. *Angewandte Chemie International Edition* **2023**, *62*, e202305251.

(7) Li, Z.; Mao, C.; Pei, Q.; Duchesne, P. N.; He, T.; Xia, M.; Wang, J.; Wang, L.; Song, R.; Ali, F. M.; et al. Engineered disorder in $CO_2$ photocatalysis. *Nature Communications* **2022**, *13*, 7205.

(8) Wang, J.; Li, S.; Zhao, J.; Liu, K.; Jiang, B.; Li, H. Boron-doped Cu-Co catalyst boosting charge transfer in photothermal carbon dioxide hydrogenation. *Applied Catalysis B: Environment and Energy* **2024**, *352*, 124045.

(9) Xu, Y.; Duchesne, P. N.; Wang, L.; Tavasoli, A.; Ali, F. M.; Xia, M.; Liao, J..; Kuang, D.; Ozin, G. A. High-performance light-driven heterogeneous $CO_2$ catalysis with near-unity selectivity on metal phosphides. *Nature Communications* **2020**, *11*, 5149.

(10) He, Z.; Li, Z.; Wang, Z.; Wang, K.; Sun, Y.; Wang, S.; Wang, W.; Yang, Y.; Liu, Z. Photothermal $CO_2$ hydrogenation to hydrocarbons over trimetallic Co–Cu–Mn catalysts. *Green Chemistry* **2021**, *23*, 5775-5785.
